# Anastomotic leakage after resection of the rectosigmoid colon in primary ovarian cancer

**DOI:** 10.1186/s13048-023-01153-x

**Published:** 2023-04-29

**Authors:** Ji Hyun Kim, Won Ho Han, Dong-Eun Lee, Sun Young Kim, Kiho You, Sung Sil Park, Dong Woon Lee, Sang-Soo Seo, Sokbom Kang, Sang-Yoon Park, Myong Cheol Lim

**Affiliations:** 1 Center for Gynecologic Cancer, Hospital, Rare and Pediatric Cancer Branch and Immuno-oncology Branch, Research Institute, Department of Cancer Control and Policy, Graduate School of Cancer Science and Policy, National Cancer Center, 323, Ilsan-ro, Ilsandong-gu, Goyang-si, Gyeonggi-do 10408 Republic of Korea; 2 Department of Critical Care Medicine, Hospital, National Cancer Center, Goyang, South Korea; 3 Biostatistics Collaboration Team, National Cancer Center, Goyang, Republic of Korea; 4 Department of Obstetrics and Gynecology, Seoul National University College of Medicine, Seoul, Korea; 5 Center for Colorectal Cancer, Hospital, National Cancer Center, Goyang, South Korea; 6 Department of Cancer Control & Population Health, National Cancer Center Graduate School of Cancer Science and Policy, Goyang, Republic of Korea; 7 Division of Clinical Research, Research Institute, National Cancer Center, Goyang, Republic of Korea; 8 Rare and Pediatric Cancer Branch and Immuno-oncology Branch, Division of Rare and Refractory Cancer, Research Institute, National Cancer Center, Goyang, Republic of Korea

**Keywords:** Ovarian cancer, Rectosigmoid resection, Anastomotic leakage

## Abstract

**Background:**

The aim of the study is to evaluate the risk factors of anastomotic leakage (AL) and develop a nomogram to predict the risk of AL in surgical management of primary ovarian cancer.

**Methods:**

We retrospectively reviewed 770 patients with primary ovarian cancer who underwent surgical resection of the rectosigmoid colon as part of cytoreductive surgery between January 2000 to December 2020. AL was defined based on radiologic studies or sigmoidoscopy with relevant clinical findings. Logistic regression analyses were performed to identify the risk factor of AL, and a nomogram was developed based on the multivariable analysis. The bootstrapped-concordance index was used for internal validation of the nomogram, and calibration plots were constructed.

**Results:**

The incidence of AL after resection of the rectosigmoid colon was 4.2% (32/770). Diabetes (OR 3.79; 95% CI, 1.31–12.69; p = 0.031), co-operation with distal pancreatectomy (OR, 4.8150; 95% CI, 1.35–17.10; p = 0.015), macroscopic residual tumor (OR, 7.43; 95% CI, 3.24–17.07; p = 0<001) and anastomotic level from the anal verge shorter than 10 cm (OR, 6.28; 95% CI, 2.29–21.43; p = 0.001) were significant prognostic factors for AL on multivariable analysis. Using four variables, the nomogram has been developed to predict anastomotic leakage: https://ALnomogram.github.io/.

**Conclusion:**

Four risk factors for AL after resection of the rectosigmoid colon are identified from the largest ovarian cancer study cohort. The nomogram from this information provides a numerical risk probability of AL, which could be used in preoperative counseling with patients and intraoperative decision for accompanying surgical procedures and prophylactic use of ileostomy or colostomy to minimize the risk of postoperative leakage.

**Trial registration:**

Retrospectively registered.

**Supplementary Information:**

The online version contains supplementary material available at 10.1186/s13048-023-01153-x.

## Introduction

Ovarian cancer is a leading cause of death in gynecologic malignancy that is frequently diagnosed at an advanced stage with peritoneal carcinomatosis [1–3]. According to cancer statistics provided by the American Cancer Society, ovarian cancer accounts for the fifth-highest mortality rate among all malignancies in the United States, with an estimation of 21,410 new cases and 13,770 death in 2021 [1]. In Korea, ovarian cancer is estimated to account for 3,173 new cases by 2022 [4], and the incidence rate is gradually increased [2].

Resection of the rectosigmoid colon is frequently required to achieve complete resection with no gross residual tumor, which is a crucial factor for improving overall survival [5, 6]. However, anastomotic leakage (AL) is a major complication that increases postoperative morbidity and mortality [7]. Postoperative AL is related to poorer perioperative outcomes, including longer length of hospital stay, delayed time to start adjuvant chemotherapy, and higher short-term mortality, which may contribute to negative survival outcomes [7–10]. To improve surgical outcomes, prevention of AL is an important issue, and preoperatively assessing risk factors and individually quantifying the risk of AL needs to be investigated.

In previous studies, predisposing factors of anastomotic leak in ovarian cancer were identified including low albumin level (< 3.0 mg/dl), old age, bevacizumab, additional bowel resection, or hand-sewn anastomosis [10–14]. However, in the majority of studies, only a single variable was identified as an independent risk factor from the relatively small study cohort, and some factors such as hand-sewn anastomosis were lack of reproducibility due to technological advances in anastomotic devices. Therefore, this study aims to determine the risk factors for AL after resection of the rectosigmoid colon and anastomosis using current standard surgical techniques and continuous variables of serum albumin in women with ovarian cancer and develop a nomogram to predict the risk of AL to use daily clinical practice.

## Materials and methods

**Fig. 1:**
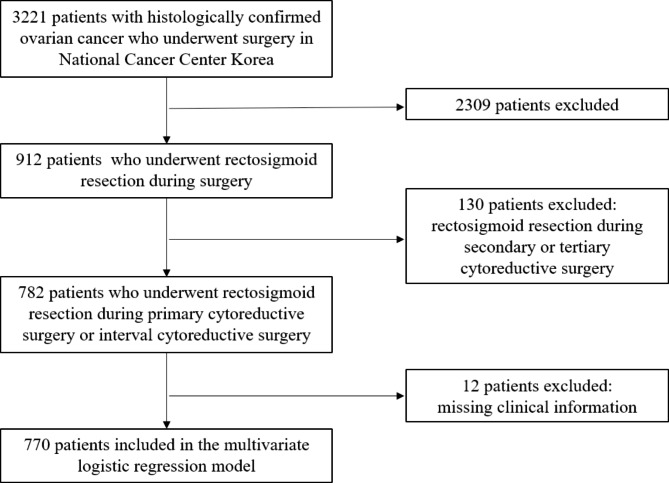
Flow diagram of the study

This study was an evaluation of retrospectively collected data from patients pathologically diagnosed with primary ovarian cancer and underwent rectosigmoid resection during cytoreductive surgery at the National Cancer Center, Korea. Patients who had undergone neoadjuvant chemotherapy and interval cytoreductive surgery were also included. Between January 2000 to December 2020, total of 770 patients were identified in the study. Patients with missing clinical data were excluded, and no imputation was used in this study (Fig. 1) The study was approved by the Institutional Review Board of our Ethics Committee.

Data including demographic information, surgical records, pathologic reports, adjuvant chemotherapy records, and administrative records were extracted by two clinicians and validated by the other two clinicians. Demographic variables such as age at surgery, comorbidities including diabetes, American Society of Anesthesiologists (ASA) score was assessed. For the preoperative nutritional assessment, preoperative serum albumin level, and body mass index (BMI) which classified into three categories; underweight patients with BMI < 18.5 kg/m^2^, normal weight with BMI 18.5–24.9 kg/m^2^, and overweight with BMI ≥ 25 kg/m^2^ were used. The histologic type, pathological stage of the International Federation of Gynecology and Obstetrics (FIGO 2014), and tumor grade were recorded.

Timing of cytoreductive surgery and details about surgical procedures were recorded as follows: omentectomy, pelvic or paraaortic lymphadenectomy, peritonectomy, cholecystectomy, distal pancreatectomy, splenectomy, colectomy, hyperthermic intraperitoneal chemotherapy (HIPEC) and large or small bowel resection and anastomosis. The records of ligation and section of the inferior mesenteric artery and anastomotic level from the anal verge was assessed. A residual tumor (RT) was classified with microscopic RT, minimal macroscopic RT of 1 cm or less, and macroscopic RT of more than 1 cm.

Postoperative complications that occurred during the period between the surgery date and completion of adjuvant chemotherapy were graded by Clavien-Dindo classification [15], and dates of anastomotic leaks that occurred during the same observation period were collected. AL was diagnosed based on radiologic studies or sigmoidoscopy with relevant clinical symptoms. Severity of anastomotic leak was classified with the International Study Group of Rectal Cancer (ISREC) criteria consisting three grades [16]. If ileostomy was made after a diagnosis of anastomotic leakage, the reversal date of stoma was also recorded. Other information included the timing of adjuvant chemotherapy and the regimen used for adjuvant and the use of bevacizumab therapy.

### Statistical analysis

Patient characteristics and postoperative features were summarized as frequencies with percentages in the case of categorical variables, and median with minimum-maximum of continuous variables. A logistic regression model was used to identify the prognostic factors of AL. Variables that were found to be risk factors for AL (P-value < 0.05) in the univariable analysis were entered to the multivariable logistic regression model. Moreover, a backward variable selection method with an elimination criterion of minimizing the Akaike information criterion (AIC) was used to fit the multivariate model. The discrimination of models was confirmed using Area Under the Receiver Operating Characteristic curve (AUC). The internal validation of a model via the bootstrap sample was used optimism-adjusted AUC. It was used to evaluate the calibration of the model using a calibration plot and Hosmer-Lemeshow test that confirms the relationship between the predicted probability of the model and the actual probability. Additionally, decision curve analysis was performed for evaluating prediction models. The prediction model was reported according to TRIPOD checklist (Supplementary Table 1). All statistical analyses were conducted using SAS software, version 9.4 (SAS Institute Inc, Cary, NC, USA.) and R software, version 4.1.2 (R Foundation for Statistical Computing, Vienna, Austria.).

## Results

### Patient characteristics

Of 770 patients with primary ovarian cancer who underwent resection of the rectosigmoid colon during cytoreductive surgery, 32 (4.2%) patients experienced anastomotic leakage until the completion of first-line adjuvant chemotherapy. Baseline demographic and clinical characteristics are summarized in Table 1. The median age at surgery was 55 years and 6.5% of patients was underweight (BMI < 18.5 kg/m^2^). Forty-four patients (5.7%) had diabetes and the median serum albumin level was 4.1 g/dL. Advanced stage ovarian cancer (89.0%, 685/770) and histologic type of high-grade serous carcinoma (77.0%, 593/770) were prevalent among the study population, while early staged population (11.0%, 85/770) who underwent rectosigmoid resection due to suspicious finding or adhesions involving rectum and adnexa were included.

**Table 1:** Patient characteristics in women with primary ovarian cancer who underwent resection of the rectosigmoid colon (N = 770)

Variables	No. (%)
**Age at surgery, years**	55 (48–63)*
Mean ± SD	55.2 ± 10.8
< 60 years	487 (63.2)
≥ 60 years	283 (36.7)
**BMI, kg/m** ^**2**^	
low < 18.5	50 (6.5)
normal: 18.5–24.9	542 (70.4)
high ≥: 25	178 (23.1)
**Diabetes**	44 (5.7)
**Serum albumin level (g/dL)**	4.1 (3.8–4.4)*
**ASA**	
1	309 (40.1)
2	414 (53.8)
3 or more	47 (6.1)
**Stage (FIGO 2014)**	
IC-IIB	85 (11.0)
III	558 (72.5)
IV	127 (16.5)
**Histology**	
High grade serous	593 (77.0)
Endometrioid	37 (4.8)
Clear cell	38 (4.9)
Mucinous	13 (1.7)
Low grade serous	2 (0.3)
Others	87 (11.3)
**The interval from operation to adjuvant chemotherapy (days)**	21 (17–27)*
**Use of Intraperitoneal chemotherapy**	
No	736 (95.6)
Yes	34 (4.4)
**Use of Bevacizumab**	
No	727 (94.4)
Yes	43 (5.6)

BMI, Body mass index; ASA, American Society of Anesthesiologists;

FIGO, the International Federation of Gynecology and Obstetrics;

SD, Standard deviation;

*Median (the interquartile range)

The median interval from operation date to initiation of adjuvant chemotherapy was 21 days. Patients who had adjuvant intraperitoneal chemotherapy were at 4.4% (34/770), and bevacizumab was used in 5.6% (43/770).

### Surgical characteristics

Patients who underwent primary cytoreductive surgery were reported in 65.6% (505/770), and accompanied surgical procedures were as detailed in Table 2. Pelvic or paraaortic lymphadenectomy was performed in 89.9% (692/770) of patients, and distal pancreatectomy was performed in 3.4% (26/770) of patients. Additional bowel resection was performed in 18.8% (145/770) patients, including total colectomy, hemicolectomy, or bowel resection and anastomosis. Diverting ileostomy or colostomy for prophylactic purpose was performed in 8.1% of patients (62/770). Complete cytoreduction with no macroscopic disease was achieved in 71.7% of patients (552/770), and residual disease less than 1 cm was in 27.1% (209/770). Two hundred and eighteen (28.3%) had any postoperative complications grade III-IV, and median estimated blood loss was 410mL.

**Table 2:** Surgical characteristics in women with primary ovarian cancer who underwent resection of the rectosigmoid colon (N = 770)

Variables	No. (%)
**Timing of cytoreductive surgery**	
Primary cytoreductive surgery	505 (65.6)
Interval cytoreductive surgery	265 (34.4)
**Accompanied surgical procedures**	
Lymphadenectomy	692 (89.9)
Omentectomy	700 (90.9)
Splenectomy	209 (27.1)
Cholecystectomy	112 (14.6)
Distal pancreatectomy	26 (3.4)
Diaphragm peritonectomy	275 (35.8)
Diverting Ileostomy	56 (7.3)
Total Colectomy or Hemicolectomy	40 (5.2)
Large or small bowel R&A	105 (13.6)
HIPEC	61 (7.9)
**Residual tumor**	
Microscopic	552 (71.7)
≤ 1 cm	209 (27.1)
> 1 cm	9 (1.2)
**Anastomotic leak**	
No	738 (95.8)
Yes	32 (4.2)
**IMA Ligation (Missing = 2)**	
No	543 (70.7)
Yes	225 (29.3)
**Anastomotic level from the anal verge, mm**	97.5 (85–110)*
**Any postoperative complications graded by Clavian-Dindo Scale**	
I-II	552 (71.7)
III-V	218 (28.3)
**Protective ostomy**	
None	708 (91.9)
Ileostomy/colostomy	62 (8.1)
**Operation time, min**	435 (344–519)*
**Blood loss, mL**	410 (500–1150)*

HIPEC, Hyperthermic intraperitoneal chemotherapy; R&A, resection and anastomosis

IMA, Inferior mesenteric artery

*Median (the interquartile range)

### Clinical presentation of anastomotic leakage

Of 32 patients with anastomotic leakage, 34.4% (11/32) patients had additional bowel surgery including ileocectomy, hemicolectomy, large or small bowel mass excision with resection of the rectosigmoid colon **(Supplementary Table **2). The median interval from initial operation to the diagnosis of AL was 22 days. 46.9% (15/32) patients were graded B requiring antibiotics management (43.8%, 14/32) or interventional drainage (0.3%, 1/32), and 53.1% (17/32) patients were graded C, managed with surgical intervention. In the surgically treated group, loop T-colostomy, ileostomy, and Hartmann’s operation were performed in 64.7% (11/32), 12.5% (4/32), and 6.3% (2/32), respectively.

### Nomogram construction

**Fig. 2:**
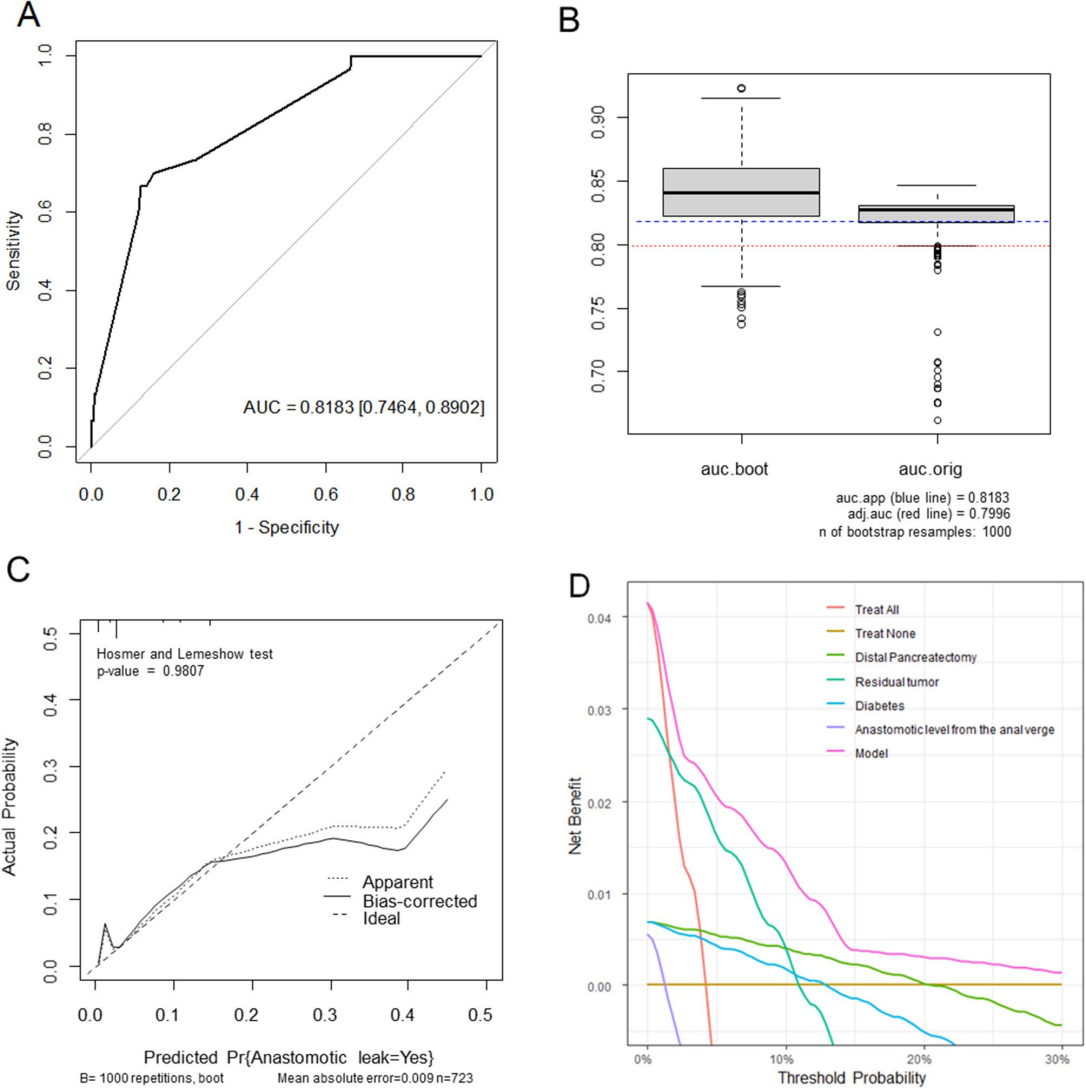
Nomogram to predict anastomotic leakage after resection of rectosigmoid colon for primary ovarian cancer

According to univariable analysis, five risk factors including diabetes (OR, 3.32; 95% CI, 1.21–9.09; p = 0.02), lower serum level of albumin (OR, 3.37; 95% CI, 1.11–10.23; p = 0.032), distal pancreatectomy (OR 6.32, 95% CI 2.22–18.04; p = 0.001), residual tumor (OR 7.12, 95% CI 3.24–15.66; p < 0.001) and anastomotic level from anal verge shorter than 10 cm (OR, 5.48; 95% CI, 1.89–15.87; p = 0.002) were associated with AL (Table 3). In multivariable analysis, the incorporation of four factors except serum level of albumin presented the smallest AIC value. Based on multivariable analysis, a nomogram was developed as presented in Fig. 2. Each risk factor is allocated a score between 0 and 100 through the points scale, and total points are ranged from 0 to 355.

**Table 3:** Risk factors associated with anastomotic leak using a logistic regression model

	Univariable analysis		Multivariable analysis	
	OR (95% CI)	p-value	OR (95% CI)	p-value
**Age at surgery**	0.999(0.967–1.032)	0.9508		
**BMI, kg/m2**				
normal: 18.5–24.9	1(ref)	(0.7488)		
low < 18.5	1.584(0.456–5.505)	0.5454		
high ≥: 25	1.168(0.508–2.684)	0.8763		
**Histology**				
High grade serous	1(ref)			
Others	0.609(0.231–1.607)	0.3166		
**Diabetes**				
No	1(ref)		1(ref)	
Yes	3.319(1.212–9.088)	**0.0196**	3.788(1.130–12.693)	**0.0309**
**Timing of cytoreductive surgery**				
PCS	1(ref)			
ICS	0.998(0.474–2.103)	0.9961		
**Serum albumin level (g/dL)**				
≥3.0	1(ref)			
<3.0	3.372(1.112–10.225)	**0.0318**		
**ASA**				
1	1(ref)	(0.0897)		
2	0.930(0.429–2.017)	0.1100		
3 or more	2.946(0.989–8.782)	0.0294		
**Stage (FIGO 2014)**				
I, II	1(ref)	(0.6604)		
III	1.946(0.453–8.371)	0.3953		
IV	1.701(0.322–8.975)	0.7366		
**Cholecystectomy**				
No	1(ref)			
Yes	1.376(0.553–3.422)	0.4925		
**Distal pancreatectomy**				
No	1(ref)		1(ref)	
Yes	6.323(2.216–18.037)	**0.0006**	4.806(1.351–17.099)	**0.0153**
**Splenectomy**				
No	1(ref)			
Yes	1.429(0.677–3.017)	0.3496		
**Residual tumor**				
Microscopic	1(ref)		1(ref)	
Macroscopic	7.116(3.237–15.645)	**<0.0001**	7.434(3.238–)	**<0.0001**
**Additional bowel resection**				
No	1(ref)			
Yes	1.348(0.571–3.183)	0.4964		
**Use of Bevacizumab**				
No	1(ref)			
Yes	0.535(0.071–4.012)	0.5425		
**HIPEC**				
No	1(ref)			
Yes	0.767(0.179–3.29)	0.7216		
**Protective ostomy**				
None	1(ref)			
Ileostomy/colostomy	2.213(0.821–5.966)	0.1164		
**Operation time, min**	1.001(0.998–1.004)	0.4469		
**Blood loss**	1.002(1.000-1.003)	0.0896		
**IMA ligation (Missing = 2)**				
No	1(ref)			
Yes	1.101(0.513–2.365)	0.8042		
**Anastomotic level from the anal verge (cm) (Missing = 47)**				
≥ 10.0	1(ref)		1(ref)	
< 10.0	5.480(1.892–15.868)	**0.0017**	7.008(2.291–21.434)	**0.0006**

OR, Odds ratio; BMI, Body mass index; ASA, American Society of Anesthesiologists;

FIGO, the International Federation of Gynecology and Obstetrics; HIPEC, Hyperthermic intraperitoneal chemotherapy; IMA, Inferior mesenteric artery

**Fig. 3:**
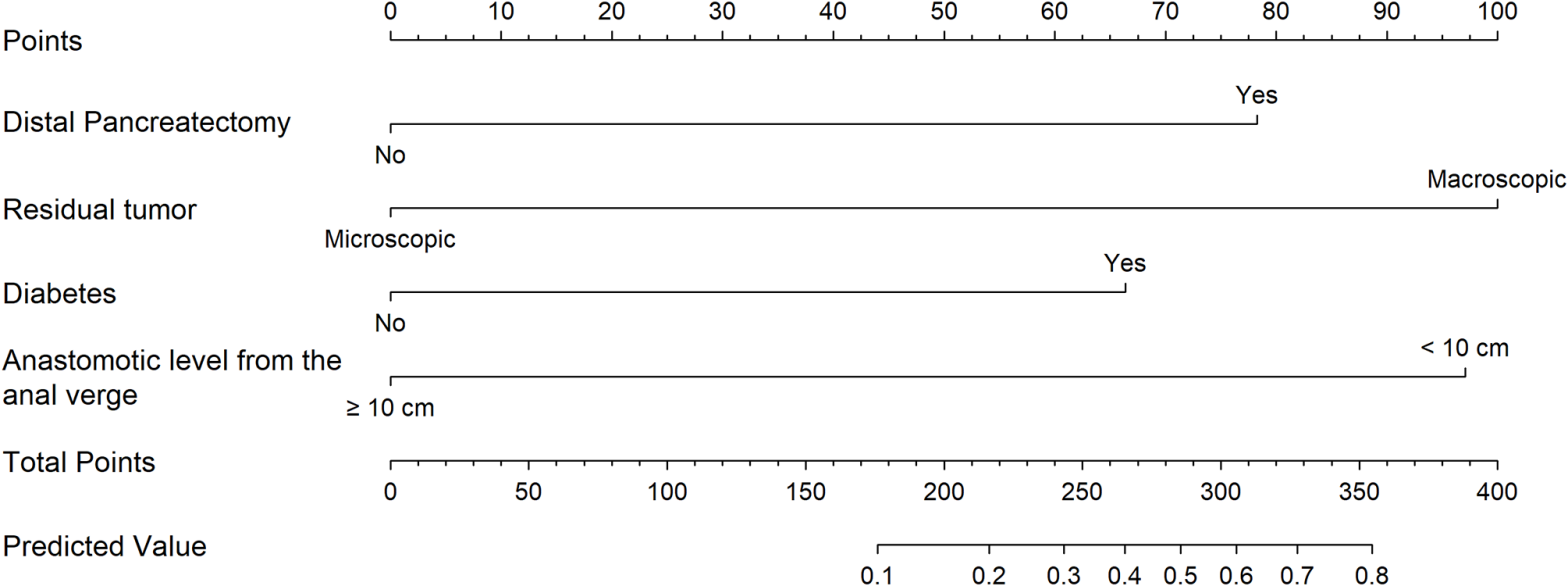
Evaluation of model performance for predicting anastomotic leak

The model for prediction achieved concordance index (c-index) of 0.818 (95% CI, 0.746–0.890, Fig. 3A) and Optimism-adjusted AUC was 0.779 (95% CI, 0.730–0.863, Fig. 3B). The P-value of the Hosmer-Lemeshow goodness of fit test was not significant, it was confirmed that the model was well calibrated (Fig. 3C). The predictive model has a relatively high net benefit over the entire threshold probability range (Fig. 3D).

For easier clinical use, the online nomogram is developed, and the link is as follows: https://ALnomogram.github.io/.

## Discussion

In this study, the rate of AL was 4.2% (32 of 770 patients), which is lower than the median rate of 5.3% in the previously reported meta-analysis of thirteen studies for ovarian cancer [17]. Of patients with AL, seventeen (53.1%) patients required re-laparotomy. This study was conducted in the largest Asian population-based cohort, to our knowledge.

The following four variables were identified to independent risk factors of AL; preoperative comorbidity (diabetes), and surgical features (co-operation of distal pancreatectomy, low anastomotic level, and macroscopic residual disease).

Corresponding with the previous studies which identified relevant risk factors of AL in ovarian cancer, low preoperative albumin level [11, 12, 18, 19] and low anastomotic level from the anal verge were resulted in the significant risk factors [9, 12, 18]. Hypoalbuminemia is a well-established risk factor of AL, that is believed to be related to being vulnerable to inflammation and inhibits tissue synthesis [20–22]. In this study, the cut-off serum albumin level was < 3.0 g/dL, which was elevated the risk of AL. anastomotic level shorter than 10 cm as was confirmed to another contributing factor to risk of AL, which was known to increase tensile strength and decrease submucosal blood flow [9, 23, 24].

In our cohort, following three independent variables were newly identified as a risk factor; diabetes, co-operation of distal pancreatectomy, and postoperative residual disease.

Because of the heterogeneous circumstances in assessing the risk of AL, diabetes remained a conflicting variable requiring careful interpretation. Diabetes is hypothesized to related with poor vascularity from increased risk of atherosclerosis and reduced ability to deal with infection [25, 26], however, there were prior results of increased postoperative outcomes with perioperative hyperglycemia in nondiabetic patients [27–30]. Still, hyperglycemia is one of the robust predisposing factors that affect the risk of AL [26, 30], and therefore careful blood glucose monitoring is required.

Distal pancreatectomy is accompanied when the tumor metastasizes to peripancreatic tissue or parenchyma of the pancreas or spleen [31, 32]. In our cohort, distal pancreatectomy was performed in 3.3% (26/770) of patients, but there are lacking data of the incidence of distal pancreatectomy in prior studies [12, 17, 18]. As surgical complexity increases, patients are at higher risk of postoperative complications. In particular, pancreatic leak occurs in approximately 30% of patients who underwent distal pancreatectomy, and the leakage of amylase into the abdomen could lead to severe infections within the abdominal cavity. [32–34]

In this context, macroscopic residual disease as a contributing factor to AL might be related to high surgical complexities. In our cohort, 89.0% of patients were advanced stage, while optimal cytoreduction was achieved in 99.7% of patients. To achieve the lowest residual disease, it is more likely to had high complexity surgery. It is also hypothesized that, residual cancer cells might yield the chance of acceleration of altering tumor microenvironment accompanied by the inflammatory reaction from subsequent cytokines release, mesothelial adhesion, and distortion of the basement membrane [35, 36]. However, further evidence is needed for these identified risk factors.

This study has several strengths. First, we implemented easily accessible web-based nomogram, which could aid the personalized preoperative counseling with patients and intraoperative decision-making about accompanying surgical procedures for distal pancreatectomy, or prophylactic diverting stomas when the AL risk outweighs the surgical benefit. Moreover, the study results were derived from the single-center in which surgical volume and procedures are consistent.

However, our study has several limitations. First, the current result is based on the analysis of the retrospective study cohort. Second, during the study period, treatment strategies have been modified, including neoadjuvant chemotherapy and targeted therapies. Lastly, we only investigated the model with internal validation at this time, due to lacking events. In near future, further study for external validation is needed to determine the reproducibility of the prediction model.

## Conclusion

In conclusion, from the largest single-center cohort, four risk factors for AL after resection of the rectosigmoid colon have been identified: diabetes, the combined surgical procedure of distal pancreatectomy, macroscopic residual disease and lower anastomotic level from anal verge. Nomogram based on the clinical data provides individualized numerical risk probability of AL. Further external validation is required for the generalized use of the nomogram.

## Electronic supplementary material

Below is the link to the electronic supplementary material.

Supplementary Material 1

## Data Availability

The datasets used in the current study are available from the corresponding author on request.

## Abbreviations

AL: Anastomotic leakage
RT: Residual tumor
AUC: Area under the receiver operating characteristic curve
